# Cutaneous Squamous Cell Carcinoma in Lynch Syndrome — An Overlooked Association

**DOI:** 10.7759/cureus.13553

**Published:** 2021-02-25

**Authors:** Vivek Moorthy, Koushik Sanku, Harjinder P Singh, Ratesh Khillan, Pathik P Patel

**Affiliations:** 1 Internal Medicine, Kasturba Medical College, Manipal, IND; 2 Hematology and Oncology, Brooklyn Cancer Care, Brooklyn, USA; 3 Internal Medicine, Gandhi Medical College, Hyderabad, IND; 4 Internal Medicine, University College of Medical Sciences, New Delhi, IND; 5 Hematology and Oncology, Kingsbrook Jewish Medical Center, New York, USA; 6 Internal Medicine, B. J. Medical College, Ahmedabad, IND

**Keywords:** lynch syndrome, muir-torre syndrome, keratoacanthoma, cutaneous squamous cell carcinoma, immunotherapy, microsatellite instability, mismatch repair

## Abstract

Lynch syndrome is an autosomal dominant disorder caused by germline mutation affecting mismatch-repair genes. Genetic testing is performed selectively. Diagnosed individuals are to undergo surveillance to detect and manage Lynch syndrome-related cancers early in the course.

Muir-Torre syndrome is a phenotypic variant of Lynch syndrome characterized by sebaceous neoplasms, keratoacanthoma, or both in addition to other Lynch syndrome-related cancers. Other neoplasms of the skin, such as squamous cell carcinoma, are not recognized as part of the Lynch syndrome tumor-spectrum. We report a case of cutaneous squamous cell carcinoma occurring in a patient with Lynch syndrome and explore some of the characteristic features and significance of this association.

## Introduction

Hereditary non-polyposis colorectal cancer (HNPCC) syndrome, now known as Lynch syndrome (LS), is an autosomal dominant condition that predisposes to visceral malignancies involving colon, rectum, endometrium, ovary, stomach, pancreas, ureter, renal pelvis, among others, due to mutations in one of mismatch repair genes (MMR) [1]. Patients/family members are clinically evaluated based on certain criteria or computational risk prediction models before confirmation with genetic testing. Due to the increased risk of cancer in the affected population, various tumor-surveillance strategies are recommended [1,2].

Muir-Torre syndrome (MTS) is a subset of LS that presents with skin lesions such as sebaceous neoplasms, keratoacanthomas (KA), or both, in addition to the visceral malignancies [3]. Other skin neoplasms, such as squamous cell carcinoma (SCC), though infrequently observed, are not considered part of the LS tumor-spectrum. We report the case of a 75-year-old man who presented with a keratoacanthoma-like skin lesion of the forehead. Genetic testing of the peripheral blood revealed an MLH1 (one of the MMR genes) germline mutation that was also detected later in the skin lesion. On histopathology, the lesion was found to be a well-differentiated SCC. The first tumor to manifest in the patient involved the external auditory canal. Interestingly, it was also an SCC. We attempt to bring attention to this lesser-known association of SCC with LS and review the diagnostic evaluation of LS and the tumor surveillance strategies recommended.

## Case presentation

**Figure 1:**
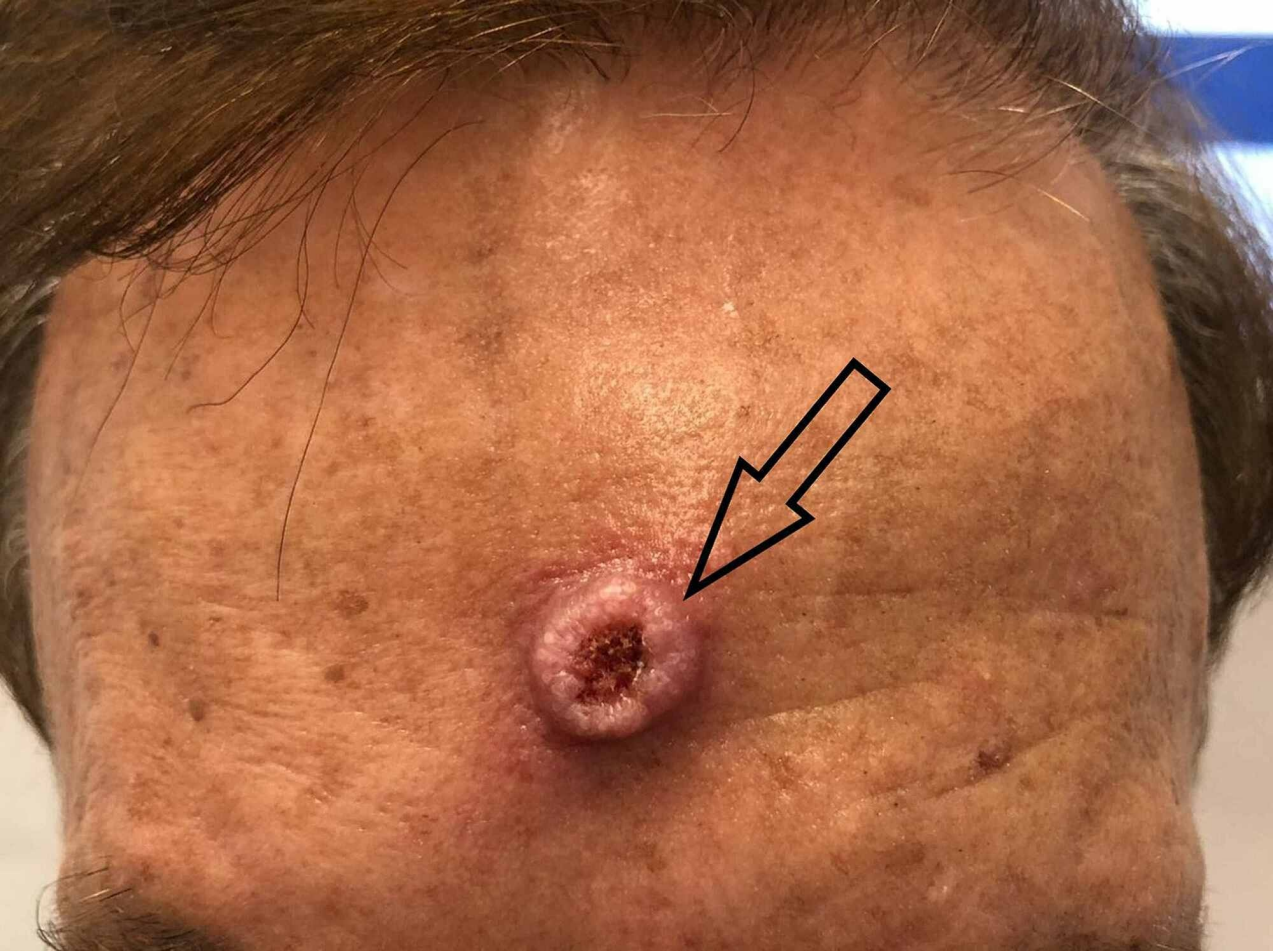
The keratoacanthoma-like presenting lesion on the forehead of the patient

A 75-year-old male patient presented to the clinic with a lesion on his forehead (Figure 1). It had started insidiously as a round papular lesion three months ago and was gradually progressing in size. Recently, it started ulcerating in the middle, leading to a crateriform center. It was non-painful, non-itchy, and without discharge. On examination, the lesion was a dome-shaped round crateriform nodule with rolled edges, measuring about 1.5 cm x 1.5 cm and 1 cm in height. Central ulceration with redness and some hyperkeratosis were noted. It had circumscribed margins with normal-appearing adjacent tissues.

At 47 years of age, he developed an SCC of the right auditory canal treated with resection and radiotherapy. Three years later, he underwent a hemicolectomy for right colon cancer, followed up every five years with colonoscopy. Both malignancies have been in remission since. The patient is a chronic hypertensive on treatment and underwent triple bypass surgery for coronary heart disease. The patient’s family history is significant for early-onset colon cancer in his mother (aged 34 years). His sister is currently undergoing treatment for colon cancer, whereas his brother had prostate cancer at 74 years.

Given his personal and family history of cancers, a sample of his peripheral blood was sent for genetic testing for common hereditary cancers (sequence analysis and deletion/duplication testing of 47 genes) (Table 1). The heterozygous pathogenic mutation (germline) of MLH1 provided molecular confirmation of LS. A possibly mosaic (somatic) pathogenic mutation of TP53 and a BRCA1 variant of uncertain significance were also detected. The keratoacanthoma-like appearance of the lesion was suspicious of the Muir-Torre variant of LS. Later, he underwent a wide excision biopsy of the forehead lesion. Histopathology revealed a well-differentiated invasive SCC with keratoacanthomatous features and actinic keratosis. Margins were cancer-free. DNA sequencing (Table 2) and immunohistochemistry (Table 3) of the tumor were performed to find any genetic mutations, microsatellite instability (MSI), and MMR deficiency. The MLH1 gene had the same pathogenic mutation as detected in the peripheral blood, which initially confirmed the diagnosis of Lynch syndrome. Microsatellite instability-high (MSI-H) and negative immunohistochemistry (IHC) for MLH1 and PMS2 were also detected. These results confirmed the SCC of the forehead as part of the LS. Several other pathogenic mutations and pathogenic variants of uncertain significance were also found in the tumor. MMR deficiency and MSI are known to cause somatic mutations/silencing [4]. For example, the specific variant of pathogenic frameshift mutation found in the BRCA1 gene (p.654fs) frequently occurs as a somatic mutation in tumors with MSI.

**Table 1:** Results of genetic testing of peripheral blood sample for common hereditary cancers The heterozygous (germline) pathogenic mutation of MLH1 provided molecular confirmation of Lynch syndrome. The TP53 mutation is likely a somatic mutation caused by mismatch repair deficiency.

Gene	Variant	Zygosity	Variant Classification
MLH1	C.866_867dup (p.Pro290Thrfs*8)	Heterozygous	Pathogenic
TP53	C.801del (p.Ash268Thrfs*77)	Possibly mosaic	Pathogenic
BRCA1	C.3785_3787del (p.Ser1262del)	Heterozygous	Uncertain significance

**Table 2:** DNA-sequencing results from the excised tumor The squamous cell carcinoma of the forehead carried the same MLH1 mutation as the germline mutation detected in the peripheral blood. Several other mutations were also detected. Mismatch repair deficiency and microsatellite instability lead to accumulation of somatic mutations that can eventually lead to tumorigenesis.

Gene	Variant interpretation	Protein alteration	Exon	DNA alteration	Variant frequency %
ASXL1	Pathogenic variant	p.G645fs	13	c.1934delG	32
BRCA1	Pathogenic variant	p.K654fs	10	c.1961delA	30
FUBP1	Pathogenic variant	p.S01fs	14	c.1201delA	23
	Pathogenic variant	p.R65fs	2	c.193delA	27
KMT2D	Pathogenic variant	p.Y2907fs	34	c.81719delT	23
MLH1	Pathogenic variant	p.P290fs	10	c.866_867dupAC	68
TP53	Pathogenic variant	p.R273C	8	c.817C>T	25
BRCA1	Variant of Uncertain Significance	p.S1262del	10	c.3785_3787delCAT	49

**Table 3:** Immunohistochemistry results of the squamous cell carcinoma of the forehead MLH1 mutations lead to loss of both MLH1 and PMS2 proteins whereas both MSH2 and MSH6 proteins are lost together in MLH2 mutation. PD-L1 positivity usually implies usefulness of immunotherapy.

Biomarker	Result
MLH1	Negative | 0
MSH2	Positive | 24, 100%
MSH6	Positive | 34, 90%
PD-L1 (SP142)	Positive | 24, 5%
PMS2	Negative | 0

## Discussion

LS is the most common inherited syndrome predisposing patients to develop colon cancer. It is an autosomal dominant disorder characterized by a germline mutation in one of several different mismatch repair genes (most commonly MLH1, MSH2, MSH6, or PMS2) or related genes that result in their suppression (e.g., EPCAM). The most commonly affected are MSH2 and MSH1 [1,5]. These mutations lead to microsatellite instability (MSI) within DNA, leading to rapidly accumulating mutations in oncogenes and tumor suppressor genes [4], eventually resulting in cancer.

Evaluation of Lynch syndrome

Universal tumor testing of all patients with colorectal cancer (CRC) would be ideal. However, in a resource-limited setting, clinical criteria such as Amsterdam II [6] and the Revised Bethesda Guidelines [7] help identify individuals at risk of Lynch syndrome (Tables 4, 5). There are few other indications as well for selective genetic testing (Table 6). This traditional or selective genetic testing involves tumor testing or germline testing based on the presence of a known mutation, clinically affected family members, and tumor samples (Table 7) [1,2].

**Table 4:** Amsterdam II clinical criteria for diagnosing Hereditary Nonpolyposis Colorectal Cancer syndrome

Amsterdam II Criteria
1. Three or more relatives with histologically verified LS-related cancers, 1 of which is a first-degree relative of the other 2. Familial adenomatous polyposis should be excluded.
2. Cancer involving at least 2 generations.
3. One or more cancer cases diagnosed before the age of 50 years.

**Table 5:** The Revised Bethesda Guidelines is an alternative clinical criterion for diagnosing Hereditary Nonpolyposis Colorectal Cancer syndrome CRC = Colorectal cancer, LS = Lynch syndrome

The Revised Bethesda Guidelines
1. CRC diagnosed at younger than 50 years.
2. Presence of synchronous or metachronous CRC or other LS-associated tumors*.
3. CRC with MSI-high pathologic-associated features (Crohn-like lymphocytic reaction, mucinous/signet cell differentiation, or medullary growth pattern) diagnosed in an individual younger than 60 years old.
4. Patient with CRC and CRC or LS-associated tumor* diagnosed in at least 1 first-degree relative younger than 50 years old.
5. Patient with CRC and CRC or LS-associated tumor* at any age in 2 first-degree or second-degree relatives.
*LS-associated tumors include tumors of the colorectum, endometrium, stomach, ovary, pancreas, ureter, renal pelvis, biliary tract, brain, small bowel, sebaceous glands, and keratoacanthomas.

**Table 6:** Indications for selective genetic evaluation

Indications for Selective Genetic Evaluation
Meets certain criteria of personal and/or family history of Lynch syndrome-related cancers (based on the Amsterdam II criteria and the Revised Bethesda guidelines)
Personal history of mismatch-repair deficient tumor
Known pathologic variant in the family
5% or higher risk for Lynch syndrome according to computational models.

**Table 7:** Selective/traditional genetic testing The selective genetic testing for confirmation of Lynch syndrome is based on the presence of a known mutation, clinically affected family members, and tumor samples.

Known family mutation	Tumor sample available from a clinically affected family member	Appropriate genetic testing strategy
Yes	Yes	Mutation-specific testing of the tumor
Yes	No	Mutation-specific germline testing
No	Yes	MSI/IHC testing of tumor tissue followed by germline testing guided by the IHC results
	No	Non-specific germline testing of MMR/EPCAM genes
No	Yes	MSI/IHC testing of tumor tissue followed by germline testing guided by the IHC results
	No	Non-specific germline testing of MMR/EPCAM genes

The patient presented above fulfilled most of the clinical criteria. Although other clinically affected family members were available, there were no known variant mutation or tumor samples available for testing. Due to high clinical suspicion and the feasibility of the genetic evaluation in the present times, genetic sequencing of peripheral blood was done for commonly affected genes in hereditary cancers. A pathogenic variant mutation of the MLH1 gene was detected, confirming LS.

Skin lesions in Lynch syndrome

Muir-Torre syndrome (MTS) is a subtype of Lynch syndrome that involves sebaceous neoplasms (e.g., adenoma, carcinoma, epithelioma), keratoacanthomas, or both in addition to other LS-related tumors. Basal cell carcinomas with sebaceous differentiation may also be seen [3]. Other skin neoplasms are not considered part of the LS/MTS tumor spectrum.

The keratoacanthoma-like skin lesion on the forehead with which the patient presented tested positive for the same MLH1 germline mutation found in the peripheral blood. MSI-H and negative MLH1 and PMS2 proteins on immunohistochemical staining were also detected. On biopsy, the lesion turned out to be a well-differentiated invasive SCC with keratoacanthomatous features. A pathological diagnosis of keratoacanthoma (KA) would have favored MTS. However, it is worth mentioning that KA has many similarities with SCC and is considered a variant of SCC by some pathologists [8].

Cutaneous squamous cell carcinoma & Lynch syndrome

The spectrum of neoplasms associated with LS is extensive. As a result, some may be overlooked and left out of a discussion on LS. One such example is cutaneous SCC. The patient presented has had three malignancies - SCC of the external auditory canal at age 47 years, right colon cancer at age 50 years, and SCC (with keratoacanthomatous features) of the forehead at age 75 years. A germline MLH1 gene mutation was detected, which was also present in the SCC of the forehead. The time passed since the first two malignancies precluded any genetic testing. However, given their age of presentation, the family/personal history of multiple cancers, and the MLH1 gene mutation in the germline and the SCC of the forehead, they are more likely part of LS than of merely sporadic nature.

Both cutaneous SCC and its precancerous lesions have been observed and reported in patients with LS since it was recognized [3,9]. Reduced MMR protein expression has also been implicated in the development of SCC from its precancerous lesions [10]. Two large observational studies investigating the subsequent primary malignancies following primary endometrial and ovarian carcinomas and the familial association of colorectal adenocarcinomas with primary malignancies at other sites found an increased risk/association with cutaneous SCC [11,12]. Endometrial, ovarian and colorectal carcinomas are notable tumors in the LS spectrum [2].

The similarities between cutaneous SCC and KA may explain the association of the former with Lynch syndrome. KAs are seen in the Muir-Torre variant of LS, and patients with multiple KAs must be asked about personal and family history of LS/LS-related cancers. Many biological and histopathological similarities and differences exist between the two lesions. The idea of KA being a type of cutaneous SCC has long been a source of debate and misdiagnosis [8,13,14]. However, the overwhelming similarities between the two may help understand where and how cutaneous SCC fits in the spectrum of LS. Moreover, since both of these lesions can have keratoacanthomatous morphology, it may be beneficial to manage keratoacanthoma-like lesions as SCC. They must be excised and sent for histopathology to be differentiated since cutaneous SCC can progress. Therefore, expectant management is best avoided despite the possibility of spontaneous resolution of KAs [5].

Certain distinctive features about cutaneous SCC associated with LS were observed in the presented case and other reported cases in the literature. Firstly, they may be one of the first manifestations of LS [15]. The SCC of the external auditory canal was the first tumor to manifest in the patient at 47 years. Therefore, a positive family or personal history of LS/LS-related cancers in a patient with newly diagnosed cutaneous SCC warrants further evaluation. Secondly, they may affect unusual sites [15]. SCC of the skin commonly affects sun-exposed areas. In the case presented, one of the two SCCs involved the external auditory canal. Thirdly, they may exhibit variable microsatellite instability. The SCC of the forehead exhibited MSI-H, but MSI-low SCC cases have also been reported previously. It is therefore recommended that MSI-low results not be classified as stable hastily and thereby preclude further evaluation. In cases of doubt, IHC is advised [3].

Tumor surveillance

The risk of cancer varies across the different MMR genes affected in terms of the site involved, the incidence/cumulative risk, and the average age of presentation. The surveillance strategies also vary to a minor extent accordingly. For example, the National Comprehensive Cancer Network (NCCN) recommendations for patients with LS due to MLH1 mutation, along with the estimated age of presentation and the cumulative risks, have been compiled in Table 8 [2]. Morbidity and mortality associated with some LS-related cancers can be lowered with early detection and management through screening [1,2]. It is therefore essential to also recognize the less common cancers in LS. Given the association of cutaneous SCC with LS, we suggest that physicians pursue the possibility of cutaneous SCC in patients with LS/MTS presenting with keratoacanthoma-like lesions.

**Table 8:** Cancer risks and average age of presentation for Lynch syndrome due to MLH1 mutation along with recommended surveillance and preventive strategies Cancer sites, their average estimated ages of presentation, and cumulative risk through 80 years vary among the different genes affected in Lynch syndrome (LS).

Cancer	Average of Presentation (years)	Cumulative Risk for Diagnosis through age 80 years (%)	Recommended Surveillance and Preventive Strategies
Colorectal	44	46-61	High-quality colonoscopy repeated every 1-2 years starting at age 20-25 years or 2-5 years prior to the earliest colon cancer in the family (if diagnosed before the age of 25 years). 600 mg Aspirin daily for at least two years decreases the risk of colorectal cancer in LS. Aspirin use should be considered on an individual basis.
Endometrial	49	34-54	Screening for endometrial cancer has not proven beneficial in LS. Endometrial biopsy every 1-2 years starting at age 30-35 years may be considered.
Ovarian	46	4-20	Since there is no proven effective screening for ovarian cancer, women should be educated on warning signs and symptoms associated with ovarian cancer.
Urothelial	59	2-7	Surveillance may be considered in select individuals such as those with family history of urothelial cancer. Surveillance options include annual urinalysis starting at age 30-35 years.
Renal Pelvis and/or Ureter	59-60	0.2-5	
Gastric	52	5-2	Consider baseline esophagogastroduodenoscopy (EGD) with random biopsy of the proximal and distal stomach for H. pylori, autoimmune gastritis, and intestinal metaplasia at age 40 years. Follow-up with surveillance EGD every 3-5 years in the presence of risk factors such as male sex, older age, MLH1 pathogenic variant, a first-degree relative with gastric cancer, Asian ethnicity, residing in or immigrant from countries with high background incidence of gastric cancer, chronic autoimmune gastritis, gastric intestinal metaplasia, and gastric adenomas.
Small Bowel	47	0.4-11	
Pancreatic	_	6.2	Consider screening starting at age 50 years (or 10 years younger than the earliest exocrine pancreatic cancer diagnosis in the family (whichever comes first) among individuals with exocrine pancreatic cancer in >1 first-or-second-degree relatives on the same side as the affected family. Consider annual contrast-enhanced MRI and/or EUS (endoscopic ultrasound). The frequency may be increased in case of any alarming findings.
Biliary Tract	50	1.9-3.7	No recommendation available as of yet
Prostate	63	4.4-11.6	Due to a lack of evidence to recommend earlier or more frequent screening for prostate cancer in men with LS, they are encouraged to undergo screening according to general guidelines.
Breast (female)	_	10.6-18.6	No evidence exists as of today to recommend additional cancer screening beyond those based on personal or family history or average-risk breast cancer screening recommendations.
Brain	_	0.7-1.7	Annual physical/neurologic examinations are recommended beginning at age 25-30 years.

Treatment implications

Immunotherapy has been shown to be more effective in MMR-deficient tumors than MMR-proficient ones [16]. It is particularly effective when the tumor exhibits MSI-H status and PD-L1 (Programmed Cell Death Ligand-1) positivity, as seen in this case [17,18]. Pembrolizumab was approved by FDA last year for use in invasive cutaneous SCC not curable by surgery or radiation [19]. Therefore, immunotherapy may be considered in the treatment of cutaneous SCC in a patient with LS, especially when refractory to surgery and radiation (the standard treatment).

## Conclusions

Lynch syndrome, an autosomal dominant hereditary disorder caused by mutations affecting mismatch-repair genes, is diagnosed by genetic testing of affected or at-risk family members identified by clinical criteria. Keratoacanthomas and sebaceous neoplasms, along with other Lynch syndrome-related malignancies, constitute Muir-Torre syndrome. Cutaneous squamous cell carcinomas are infrequently observed in patients with Lynch syndrome. In some cases, they may be the initial manifestation and present at unusual or less common sites. The microsatellite instability statuses of these lesions may be variable. The similarities between keratoacanthomas and cutaneous squamous cell carcinoma may explain the latter's lesser-known association with Lynch syndrome.

Physicians must elicit family or personal history of Lynch syndrome or related cancers in a patient with keratoacanthoma-like lesions, especially in younger patients and when multiple. These lesions must be excised entirely with tumor-free margins. They must also pursue the possibility of squamous cell carcinoma in patients with Lynch syndrome/Muir-Torre syndrome presenting with keratoacanthoma-like lesions. Immunotherapy may be considered in treating tumors such as MSI-H (microsatellite instability-high) invasive cutaneous SCC of the skin not curable by surgery or radiotherapy.

## References

[1] Giardiello FM, Allen JI, Axilbund JE (2014). Guidelines on genetic evaluation and management of Lynch syndrome: a consensus statement by the US Multi-Society Task Force on colorectal cancer. Gastroenterology.

[2] (2021). NCCN Guidelines Genetic/Familial High-Risk Assessment: Colorectal Version 1.2020. https://www.nccn.org/professionals/physician_gls/pdf/genetics_colon.pdf..

[3] Kientz C, Joly M-O, Faivre L (2017). A case report of Muir-Torre syndrome in a woman with breast cancer and MSI-Low skin squamous cell carcinoma. Hered Cancer Clin Pract.

[4] Cox VL, Saeed Bamashmos AA, Foo WC, Gupta S, Yedururi S, Garg N, Kang HC (2018). Lynch syndrome: genomics update and imaging review. Radiographics.

[5] Nishizawa A, Nakanishi Y, Sasajima Y, Yamazaki N, Yamamoto A (2006). Muir-Torre syndrome with intriguing squamous lesions: a case report and review of the literature. Am J Dermatopathol.

[6] Vasen HF, Watson P, Mecklin JP, Lynch HT (1999). New clinical criteria for hereditary nonpolyposis colorectal cancer (HNPCC, Lynch syndrome) proposed by the International Collaborative group on HNPCC. Gastroenterology.

[7] Umar A, Boland CR, Terdiman JP (2004). Revised Bethesda Guidelines for hereditary nonpolyposis colorectal cancer (Lynch syndrome) and microsatellite instability. J Natl Cancer Inst.

[8] Manstein CH, Frauenhoffer CJ, Besden JE (1998). Keratoacanthoma: is it a real entity?. Ann Plast Surg.

[9] Hatta N, Takata A, Ishizawa S, Niida Y (2015). Family with MSH2 mutation presenting with keratoacanthoma and precancerous skin lesions. J Dermatol.

[10] Liang SB, Furihata M, Takeuchi T, Sonobe H, Ohtsuki Y (2001). Reduced human mismatch repair protein expression in the development of precancerous skin lesions to squamous cell carcinoma. Virchows Archiv.

[11] Hemminki K, Aaltonen L, Li X (2003). Subsequent primary malignancies after endometrial carcinoma and ovarian carcinoma. Cancer.

[12] Hemminki K, Chen B (2004). Familial association of colorectal adenocarcinoma with cancers at other sites. Eur J Cancer.

[13] Gleich T, Chiticariu E, Huber M, Hohl D (2016). Keratoacanthoma: a distinct entity?. Exp Dermatol.

[14] Gibbons M, Ernst A, Patel A, Armbrecht E, Behshad R (2019). Keratoacanthomas: a review of excised specimens. J Am Acad Dermatol.

[15] Khaddour K, Fields RC, Ansstas M, Rosman IS, Ansstas G (2020). Metachronous cutaneous squamous cell carcinoma in a young patient as the only presenting symptom to uncover Lynch syndrome with MLH1 Germline mutation. Hered Cancer Clin Pract.

[16] Nebot-Bral L, Coutzac C, Kannouche PL, Chaput N (2019). Why is immunotherapy effective (or not) in patients with MSI/MMRD tumors?. Bull Cancer (Paris).

[17] Ribas A, Hu-Lieskovan S (2016). What does PD-L1 positive or negative mean?. J Exp Med.

[18] Dudley JC, Lin M-T, Le DT, Eshleman JR (2016). Microsatellite instability as a biomarker for PD-1 blockade. Clin Cancer Res.

[19] (2021). FDA approves pembrolizumab for cutaneous squamous cell carcinoma. https://www.fda.gov/drugs/drug-approvals-and-databases/fda-approves-pembrolizumab-cutaneous-squamous-cell-carcinoma.

